# Supplementary figures and images for: Investigation of bacterial community and histamine production in fresh mackerel at low temperature storage
Source: PLoS One. 2025 Sep 16;20(9):e0331331. doi: 10.1371/journal.pone.0331331 (PMC12440185; doi:10.1371/journal.pone.0331331)

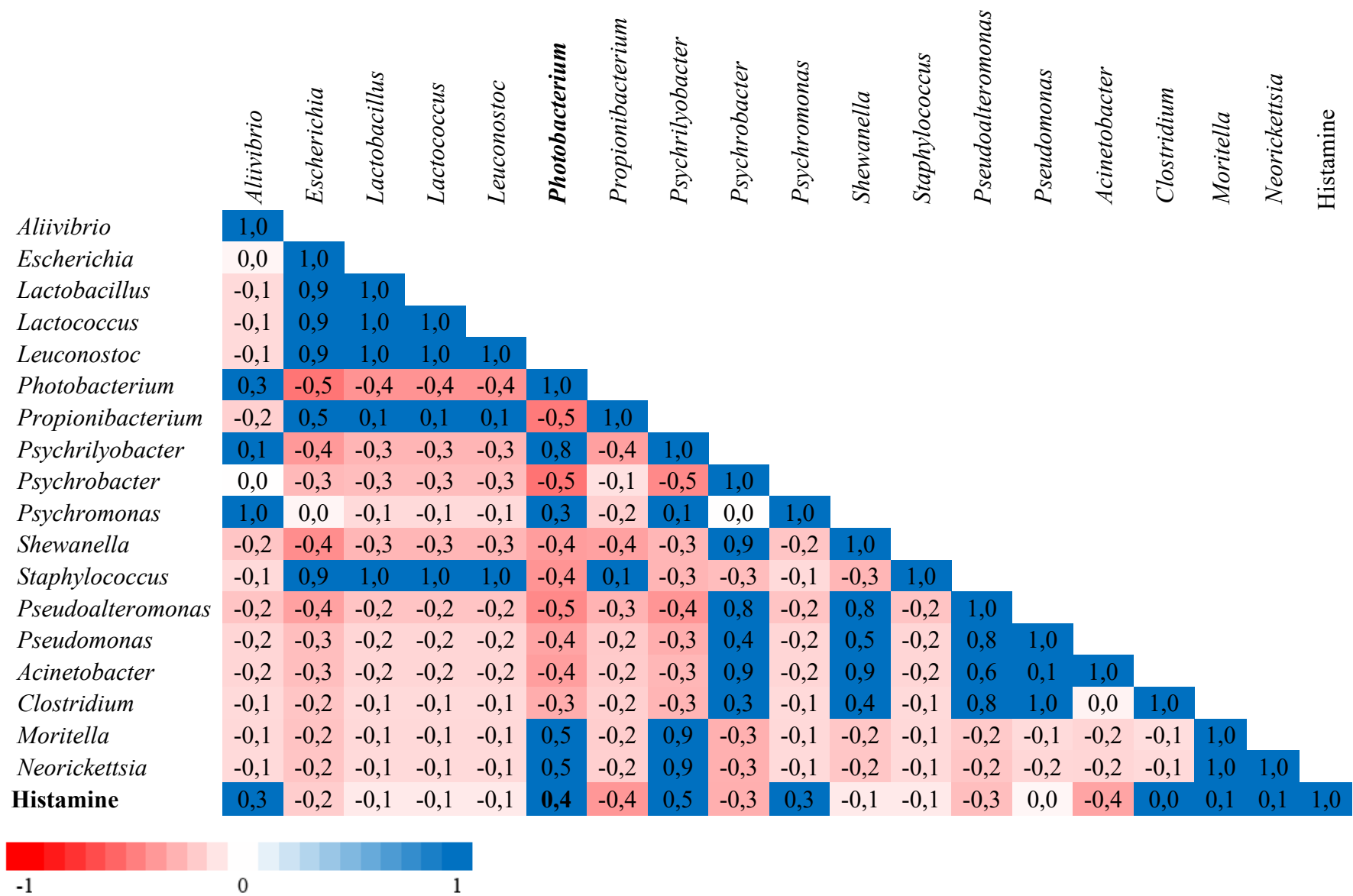

Supplement: S1 Fig — Correlation matrix of 18 significantly abundant genera and histamine level. The matrix displays positive and negative correlation between genera and histamine accumulation, represented by red and blue colors, respectively. Correlation values are normalized between –1 and +1, as indicated by the color scale bar below. (PDF) [file pone.0331331.s001.pdf]
