# Supplementary material for: Investigation of bacterial community and histamine production in fresh mackerel at low temperature storage
Source: PLoS One. 2025 Sep 16;20(9):e0331331. doi: 10.1371/journal.pone.0331331 (PMC12440185; doi:10.1371/journal.pone.0331331)
Supplement: Attachment — Submitted filename: General Comments.docx [file pone.0331331.s002.docx]

**General Comments**

**Line 70:** The manuscript does not provide the number of mackerel samples used in the study and does not specify how they were distributed among storage temperature and time groups. Information on sample size and replication is critical for assessing experimental design, statistical validity, and reproducibility.

**Line 79:** The authors state that total viable counts were performed with TSA agar. However, it is not clear from the text whether mesophilic, psychrophilic or histamine producing bacteria (HPB) were counted. It is stated that “HPB colonies were observed” but this is not supported by a specific identification method.

The incubation temperatures used are complicated and unclear: Incubation temperatures are given on TSA agar: 4°C, 20°C, 30°C. However, which group of bacteria do they target? For example: 4°C → psychrophilic 20–30°C → mesophilic

This relationship is not explained and it is not stated why these temperatures were chosen.

No selective media for histamine-producing bacteria (HPB): If HPB identification is to be made, selective media such as Niven’s agar, decarboxylase media would be required.

HPB cannot be identified with TSA alone, this is scientifically incorrect.

**Line 95-120, for all material and methods**: PCR/amplicon sequencing part: "Amplicon sequencing" was performed, but which gene region was targeted (16S V3-V4 is understood from the table, but it is not clear in the text), which primer set, which control samples were used, where is the raw data? The reader cannot clearly understand which bacteria were defined as HPB and on what basis. The methodology used for counting psychrophilic and mesophilic bacteria is not clear. TSA + ASW environment and temperatures are given, but which bacterial groups were classified as psychrophilic/mesophilic and on what basis is not explained.

HPLC method: Reference is provided, but separation conditions, detector wavelength, calibration curves, sample preparation steps are quite superficial.

**Aim:** The novelty aspect is not emphasized enough: How is this study different from previous studies on histamine production? For example, was a new HPB strain identified at the metagenomic level?

**Statistical analysis**: The statistical analysis is limited to ANOVA. Correlation analyses between microbial diversity and histamine levels could have been performed. Moreover, the type of variance analysis applied is not specified in the manuscript.

**Figures and tables:** The figures and tables are overly simplistic and not reader-friendly. For instance, Figure 2 presents only genus-level relative abundances; there is no specific analysis at the species level or of HPB carrying the hdc gene.

The manuscript should be thoroughly revised and rewritten by incorporating the above suggestions to present a clear, methodologically, and scientifically valuable study. Without these substantial corrections, the manuscript remains too ambiguous, inconsistent, and incomplete to be considered for publication in its current form.
